# Hypothermia Modulates Cytokine Responses After Neonatal Rat Hypoxic-Ischemic Injury and Reduces Brain Damage

**DOI:** 10.1177/1759091414558418

**Published:** 2014-11-21

**Authors:** Xiangpeng Yuan, Nirmalya Ghosh, Brian McFadden, Beatriz Tone, Denise L. Bellinger, Andre Obenaus, Stephen Ashwal

**Affiliations:** 1 Department of Pediatrics, Loma Linda University, CA, USA; 2 Department of Biological Sciences, California State University, Fullerton, CA, USA; 3 Department of Pathology and Human Anatomy, Loma Linda University, CA, USA; 4 Cell, Molecular, and Developmental Biology Graduate Program, Neuroscience Graduate Program, University of California, Riverside, CA, USA

**Keywords:** chemokines, cytokines, hypothermia, inflammation, MRI, neonatal ischemia

## Abstract

While hypothermia (HT) is the standard-of-care for neonates with hypoxic ischemic injury (HII), the mechanisms underlying its neuroprotective effect are poorly understood. We examined ischemic core/penumbra and cytokine/chemokine evolution in a 10-day-old rat pup model of HII. Pups were treated for 24 hr after HII with HT (32℃; *n* = 18) or normothermia (NT, 35℃; *n* = 15). Outcomes included magnetic resonance imaging (MRI), neurobehavioral testing, and brain cytokine/chemokine profiling (0, 24, 48, and 72 hr post-HII). Lesion volumes (24 hr) were reduced in HT pups (total 74%, *p* < .05; penumbra 68%, *p* < .05; core 85%, *p* = .19). Lesion volumes rebounded at 72 hr (48 hr post-HT) with no significant differences between NT and HT pups. HT reduced interleukin-1β (IL-1β) at all time points (*p* < .05); monocyte chemoattractant protein-1 (MCP-1) trended toward being decreased in HT pups (*p* = .09). The stem cell signaling molecule, stromal cell-derived factor-1 (SDF-1) was not altered by HT. Our data demonstrate that HT reduces total and penumbral lesion volumes (at 24 and 48 hr), potentially by decreasing IL-1β without affecting SDF-1. Disassociation between the increasing trend in HII volumes from 48 to 72 hr post-HII when IL-1β levels remained low suggests that after rewarming, mechanisms unrelated to IL-1β expression are likely to contribute to this delayed increase in injury. Additional studies should be considered to determine what these mechanisms might be and also to explore whether extending the duration or degree of HT might ameliorate this delayed increase in injury.

## Introduction

Based on translational studies and clinical trials, hypothermia (HT) has proven to be modestly effective in treating term and near-term neonates with moderate and to some degree severe hypoxic ischemic injury (HII; Shankaran et al., 2012). Although clinical trials are in progress to further improve outcomes, additional translational studies using HT with other treatments are warranted, as there are no consistent experimental approaches that combine imaging with cytokines and chemokines involved in injury progression.

As neuroinflammation plays a major role in postneonatal HII evolution, we concentrated our investigation on specific cytokines/chemokines known to be activated after neonatal HII and examined their association with HT and magnetic resonance imaging (MRI)-derived core and penumbral evolution (Vexler and Yenari, 2009; McAdams and Juul, 2012). Increased plasma and cerebrospinal fluid cytokine expression, particularly interleukin-1β (IL-1β) and tumor necrosis factor-α (TNF-α), occurs in term HII newborns (Clarkson et al., 2005; Aly et al., 2006) as well as in neonatal rodent models of focal but not necessarily global HII (Ashdown et al., 2008). In newborn lambs, cytokines are synthesized peripherally early after HII (i.e., 1–3 hr) but mainly as part of the central microglial neuroinflammatory response with a second peak at 24 to 36 hr (Alonso-Alconada et al., 2012). Cytokines increase other components of the inflammatory response, leading to neuronal and oligodendroglial cell death and impaired myelin synthesis. Several recent studies also suggest dual proinflammatory neurotoxic and anti-inflammatory reparative roles of cytokines (McAdams and Juul, 2012).

On the basis of a recent report by Lee et al. (2010), we have constructed an eight-compartment HT chamber that allows individual cooling of rat pups, which prevents warming effects associated with maternal huddling (Lee et al., 2010). We used MRI and a computational analysis method, Hierarchical Region Splitting (HRS), to measure ischemic core and penumbra (CP) volumes at 0, 24, 48, and 72 hr post-HII/48 hr post-HT (Ghosh et al., 2012). We also measured selected cytokines (IL-1β, TNF-α, interferon-γ [IFN-γ]) and the cytokine/chemokine monocyte chemoattractant protein-1 (MCP-1), known to participate in postneonatal HII inflammatory toxicity and one of the chemokine stem cell migrational signaling molecules (SDF-1) to determine whether they were differentially regulated by HT. We tested our hypothesis that HT would reduce inflammatory cytokine expression leading to decreased lesion volumes early after neonatal HII. Our goals were to determine (a) to what extent HT reduces lesion volumes (total, core, and penumbral) and to quantify the degree of tissue salvageability, (b) whether specific cytokine levels correlate with the injury volume and are altered by HT, and (c) whether the activities of signaling molecules that serve as chemoattractants for stem cells are modified by HT.

## Materials and Methods

**Figure 1.**
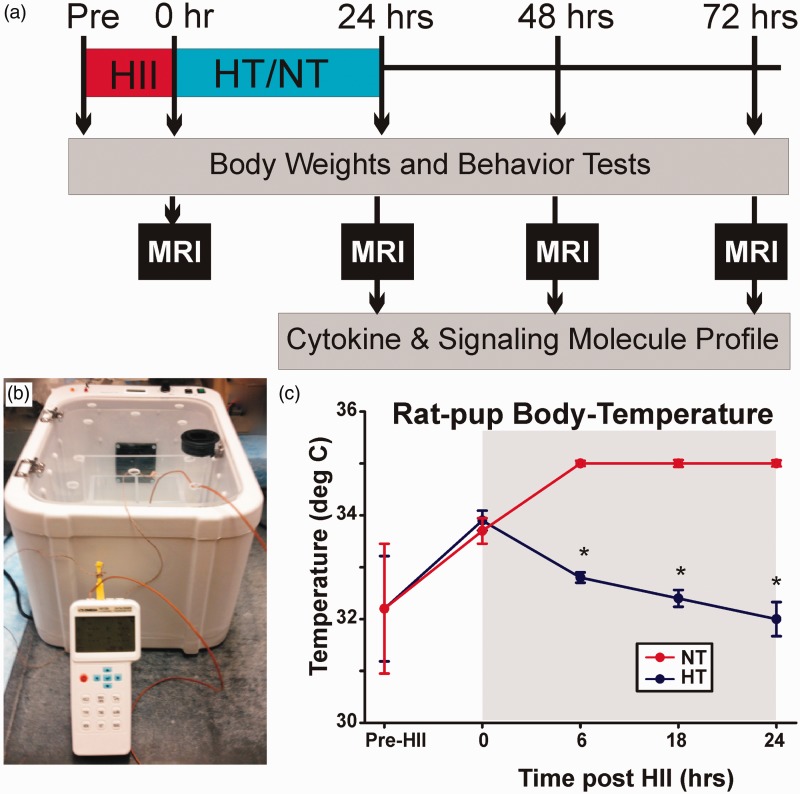
(a) Experimental timeline: Injury severity was classified using RPSS from DWI at 0 hr post-HII. Moderately injured pups then underwent HT or NT treatment for 24 hr. Body weight, neurological scores, and MRI were measured at different time points before harvesting brain tissue for cytokine/chemokine and signaling molecule profiling. (b) Hypothermia/Normothermia Chambers: Temperature-controlled chambers (HT: 30℃; NT: 35℃) were used with matrix separation for each pup. (c) Animal temperature: HT pups’ body temperatures (∼32℃) were significantly lower than NT pups (∼35℃). HII = hypoxic ischemic injury; HT = hypothermia; NT = normothermia; MRI = magnetic resonance imaging.

All experimental protocols complied with federal and Loma Linda University Animal Care and Use Committee regulations. Our experimental protocol is outlined in Figure 1(a).

### Animal Model of HII

HII of the left hemisphere was induced in 10-day-old Sprague-Dawley male rat pups (Harlan Laboratories, Livermore, CA, USA) using a modified Rice-Vannucci model (RVM) of permanent unilateral common carotid artery occlusion followed by hypoxia as previously reported (Obenaus et al., 2011). Briefly, the left common carotid artery was exposed and permanently ligated (4% isoflurane in a mixture of 40% oxygen and 60% air), and the pup was allowed to recover for 2 hr with the dam. Hypoxia was induced by placing all pups (*n* = 10 or 12) in one litter in an airtight 2,000-ml Erlenmeyer flask containing a humidified hypoxic gas mixture (8% O_2_, 92% N_2_) for 2 hr and maintained at 37℃. Animals were then allowed to recover for 15 min with the dam prior to the first neuroimaging time point at 0 hr post-HII. Animals were weighed before HII, before HT/NT, and then at each neuroimaging time point.

### HT and Normothermia Induction

**Figure 2.**
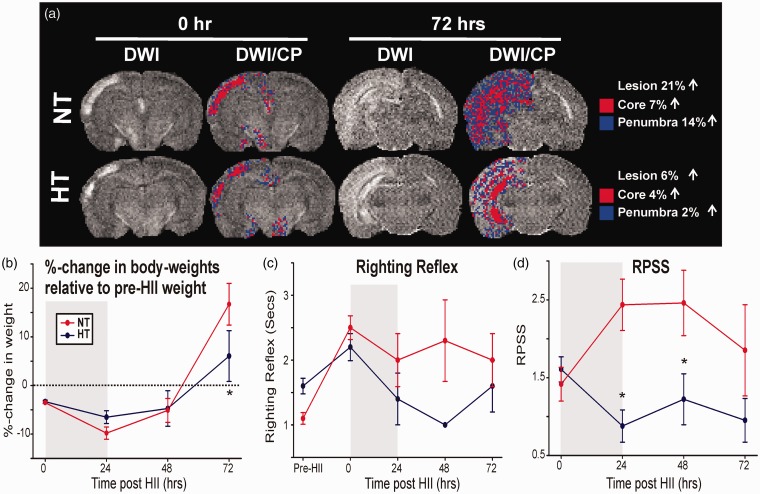
(a) MRI- and HRS-derived core and penumbra: DWI from a NT and HT pup at two time points post-HII: 0 hr (pre) and 72 hr (post) treatment. CP regions were derived by HRS, superimposed on DWI and %-volumes (of total brain) were computed. (b) Body weight changes after HT/NT treatment: Weight changes relative to the pre-HII body weight are plotted for time points before and after treatments (gray). (c) Neurological assessment (righting reflex): Righting reflex times in HT pups were significantly shorter than in NT pups after treatment. (d) RPSS, an MRI-derived measure of severity, was lower in HT compared with NT pups (*p* < .05) after treatment. HT = hypothermia; NT = normothermia; DWI = diffusion weighted imaging; CP = core and penumbra; HII = hypoxic ischemic injury; RPSS = rat pup severity score.

Diffusion weighted imaging (DWI) data were collected at 0 hr post-HII (Figure 2(a)) to compute a rat pup severity score (RPSS) to qualitatively assess HII severity (Recker et al., 2009). Out of total six litters (*n* = 70) of male pups used for this study, 10 pups died after hypoxia (mortality: 14%), 18 pups had no injury, 7 pups had mild injury (RPSS ≤ 0.25), and 2 pups had severe injury (RPSS ≥ 3.0). The remaining pups with a moderate brain injury (0.25 < RPSS < 3.0 at 0 hr; *n* = 33) were used for this study and randomly assigned to one of two treatment groups: (a) HT or (b) NT (Table 1). All pups were placed in a temperature-controlled chamber (Harvard Apparatus, Holliston, MA, USA; Figure 1(b)) and separated from each other via an acrylic lattice (3 in. × 4 in. × 4 in.) to prevent huddling. Chamber temperatures were set to 30℃ for the HT pups and 35℃ for the NT pups. Rectal temperatures were measured every 6 hr to confirm body temperatures where HT animals (∼32℃) were consistently maintained lower than those in NT animals (∼35℃; Figure 1(c)). As per our animal care facility protocol, pups were fed 0.4 mL of commercially available Similac formula every 4 hr during the 24-hr HT/NT period using a 22G-animal gavage feeding tube with a rounded tip. After 24 hr, HT/NT intervened pups were returned to their dams at normal room temperature for DWI, neurological testing, and cytokine/chemokine expression analysis (Figure 1(a)).

**Table 1.** Number of Animals at Each Time Point.

Treatment	24 h	48 h	72 h	Total
Hypothermia	6	6	6	18
Normothermia	5	5	5	15
Total	11	11	11	33

### Neuroimaging

#### MRI acquisition

Pups underwent DWI to identify and quantify the extent of brain injury at 0, 24, 48, and 72 hr post-HII (Figure 1(a)). Pups were lightly anesthetized (isoflurane 3% induction, 1% maintenance), and DWI data were collected from a Bruker Advance 11.7 T MRI scanner (8.9-cm bore) or a Bruker 4.7 T MRI scanner (30.0 cm bore) with a 3.0-cm (internal diameter) volume quadrature coil (Bruker Biospin, Billerica, MA), as previously reported (Obenaus et al., 2011). Two scanners were required to image the large number of pups at each time point (10–12 animals). DW images (Figure 2(a)) were collected using the following parameters: recovery time/echo time (TR/TE): 1,097/50 (11.7 T), 3,000/50 ms (4.7 T); *b*-values: 1044.42 (11.7 T), 441.99 s/mm^2^ (4.7 T); matrix: 128 × 128; field-of-view: 2 cm × 2 cm; with two averages. Each sequence collected 20 coronal slices (1-mm thickness, contiguous) spanning the entire brain. DWI data were processed for apparent diffusion coefficient maps (ADC).

#### MRI analysis

Data were processed using two methods that we have developed: (a) RPSS and (b) HRS (Recker et al., 2009; Ghosh et al., 2012).

##### RPSS

The RPSS is a qualitative previously-validated method to rapidly visually assess HII and was computed for each pup at every imaging time point (Recker et al., 2009). Regional (cortex/basal-ganglia/hippocampus, dorsal/ventral) scores of “1” (abnormal DWI signal) or “0” (normal tissue) were assigned, totaled for each imaging section, and normalized to the total number of MRI slices to generate a final RPSS.

##### HRS

Automated HRS was utilized for all MRI data analyses, as our previous studies established that MR-derived core and penumbral lesion volumes colocalized with those determined by immunohistochemistry or diffusion-perfusion mismatch (Ghosh et al., 2012). In brief, HRS takes a MR image (scanned image or computed map) as input, computes a global histogram of image values, fits the histogram as a bimodal distribution, finds the valley in between two distinct peaks (modes), and segments the MR image into two subimages using the valley point (adaptive threshold). Then, HRS continues this binary splitting iteratively on these subimages to form a bipartite tree of subimages (called the HRS tree). As one goes down the HRS tree branches, subimages become smaller and with more uniform MR values characterizing similar brain tissue densities in that region. Thus, ischemic injury is separated first from the normal healthy tissue, and then the injured region is further bifurcated to delineate ischemic core from penumbra using mean MR values of the regions. The HRS parameters used in the present study to dichotomize the ischemic lesion into CP that are not visually separable are detailed in Table 2. When ADC data suffered from ambiguity (e.g., at time points of diffusion reversal or when motion-related artifacts affected the ADC), we reverted to DWI scans instead of, or in combination with, the ADC data. Regional data computation included volumes (in ml and percentage of total brain) and mean ADC values of normal appearing brain matter, total lesion, core, and penumbra.

**Table 2.** DWI HRS Parameters Utilized for Automated Lesion, Core, and Penumbra Detection.

Scanner	ADC ranges (10^−6 ^mm/s^2^)				Smallest detected area (pixels)
	Normal	Lesion	Penumbra	Core	
11.7 T (Dark)	600–1,000	<550	475–550	<475	25
11.7 T (Bright)		>1,200	1,200–1,275	>1,275	25
4.7 T (Dark)	800–1,300	<700	625–700	<625	25
4.7 T (Bright)		>1,500	1,500–1,575	>1,575	25

*Note.* ADC = apparent diffusion coefficient maps.

### Neurological Testing

Motor function was assessed using the righting reflex of each rat pup at all time points (Figures 1(a) and 2(c)) by an experimenter blinded to the treatment group of the pups. The righting reflex is the time required for a rat pup to return to a prone position after being placed on their backs by the experimenter.

### Cytokine/Chemokine Determination

After the final MRI, rat pups (at 24, 48, and 72 hr post-HII; Figure 1(a), Table 1) were deeply anesthetized, their brains removed, and the ischemic injured hemispheres dissected and frozen at −80℃ for later processing. Brain tissue was homogenized in a glass homogenizer with extraction buffer (20 mmol/L Tris-HCl, pH: 7.5, 150 mmol/L NaCl, 1% Triton X-100; 1 mmol/L ethylenediaminetetraacetic acid, 1 mmol/L ethyleneglycoltetraacetic acid, 2.5 mmol/L pyrophosphate, 1 mmol/L β-glycerophosphate) containing protease and phosphatase inhibitor cocktails (Roche Applied Science, Indianapolis, IN, USA). The homogenate was centrifuged at 10,000 × g for 20 min, and the supernatant frozen at −80℃ for later cytokine and signaling molecule quantification. Multiplexed magnetic bead-based immunoassay kits (Milliplex #RECYTMAG-65 K, EMD Millipore, Billerica, MA) were used to detect IL-1β, TNF-α, IFN-γ, and MCP-1 following the manufacturer’s instructions with three exceptions. A twofold increase in sample volume was used, and an additional fourfold diluted standard was added to increase sensitivity, as recommended by the manufacturer. The mean fluorescence intensity values were divided by 2 in the data analyses to adjust for the increased sample volume. SDF-1α was detected with a separate Millipore Simplex kit. Briefly, 25 µl (50 µl for multiplexed kits) of brain tissue protein in protein extraction buffer were added to the cocktail of anti-cytokine antibody tagged with fluorescently labeled, color-coded microspheres in 96-well plates and incubated at 4℃ overnight. Subsequently, the plates were washed, and 25 µl of biotinylated detection antibodies were added to each well and the plates were incubated with agitation for 1 hr at room temperature. Finally, 25 µl of streptavidin-phycoerythrin conjugate were added to each well, and the plate was incubated for 30 min. Following incubation, the plates were washed, and then read on Magpix (Luminex Corp., Austin, TX). The data were analyzed using MasterPlex 2010 software (Hitachi Solutions America, San Bruno, CA).

### Statistical Analyses

All data are represented as the mean ± standard error. All temporal data were analyzed using a two-way ANOVA examining both repeated measure temporal and group effects (Sigma Plot V11, Systat Software Inc., San Jose, CA). Injury severity measures (RPSS- and HRS-based %-lesion volumes) were correlated for cross-validation of both measures with linear regression lines fit, and Spearman coefficient *r*^2^ values were computed. Tests were considered statistically significant at *p* values < .05. Expression levels of cytokines/chemokines were cross-correlated with HRS based %-lesion, %-core and %-penumbra volumes computed from the MRI data at that time. Linear regression coefficients (Spearman *r*^2^ values) were computed.

## Results

### HT/NT Treatment

All neonatal pups with moderate HII survived the HT/NT 24-hr period. Body temperatures for the HT (∼32℃; *n* = 18) and NT (∼35℃; *n* = 15) groups were consistently maintained over the 24-hr period and were significantly different between groups during treatment (*p* < .05; group-wise and time; Figure 1(c)). Relative to pre-HII weight, both groups lost similar amounts of weight by the end of HII (Figure 2(b)), that is, at the start of HT/NT treatment (HT: 3.3%; NT: 3.5%; *p* = .91). By the end of treatment, HT pups had a trend of less weight loss (33.5%) than NT-treated pups (HT: 6.5%, NT: 9.8%; *p* = .09). Between 48 and 72 hr, NT pups had a weight gain of 22% while HT pups gained only 11% (*p* < .05, Figure 2(b)).

### Motor Impairment

For all time points combined, the righting reflex times (RRTs) of HT pups were significantly faster than NT pups (*p* < .05, Figure 2(c)), but post-hoc testing showed no significant differences at individual time points. At 0 hr post-HII, RRTs were similar in both groups (HT: 2.2 ± 0.8 s, NT: 2.5 ± 0.7 s; *p* = .18). After treatment, HT pups demonstrated a tendency toward faster RRTs than NT pups (43%; HT: 1.4 ± 0.7 s, NT: 2.0 ± 1.0 s; *p* = .2). At 72 hr post-HII (48 hr post-HT), there was no significant differences between HT/NT groups (*p* = .40).

### Neuroimaging

#### RPSS

Semiquantitative inspection (Figure 2(d)) of the DWI scans (Figure 2(a)) using the RPSS (Recker et al., 2009) demonstrated reduced lesion size in HT compared with NT pups at all the time points (*p* < .05). Although similar at 0 hr post-HII (pretreatment; *p* = .56), the RPSS in HT pups was reduced 64% compared with NT pups at the end of the treatment (24 hr post-HII; *p* < .05), remained significantly low up to 48 hr (*p* < .05), and showed a tendency to remain low at 72 hr (*p* = .13) consistent with reduced HII severity in HT pups (Figure 2(d)). Analysis of individual pups demonstrated some heterogeneity in RPSS, where decreased RPSS was found in 70% of HT pups, while it increased in 71% of NT pups. At 24 hr post-HII (i.e., immediately post-HT/NT), the RPSS was decreased in 83% of HT pups, whereas it was increased in 87% NT pups. Between 0 hr and 72 hr post-HII, there was a 31% increase in RPSS for NT pups and a 41% decrease in RPSS for HT pups.

#### Lesion volumes

Figure 2(a) shows representative MRI images at 24 hr (i.e., onset of HT vs. NT) and at 72 hr after HII. Pretreatment HII volumes were similar in both groups (∼2%), but the total lesion volume (core + penumbra) increased to a greater extent in NT (∼21%) than in HT pups (∼6%). In these pups, HT reduced core (NT, 7%; HT, 4%) and penumbral (NT, 14%; HT, 2%) volumes at 72 hr.

**Figure 3.**
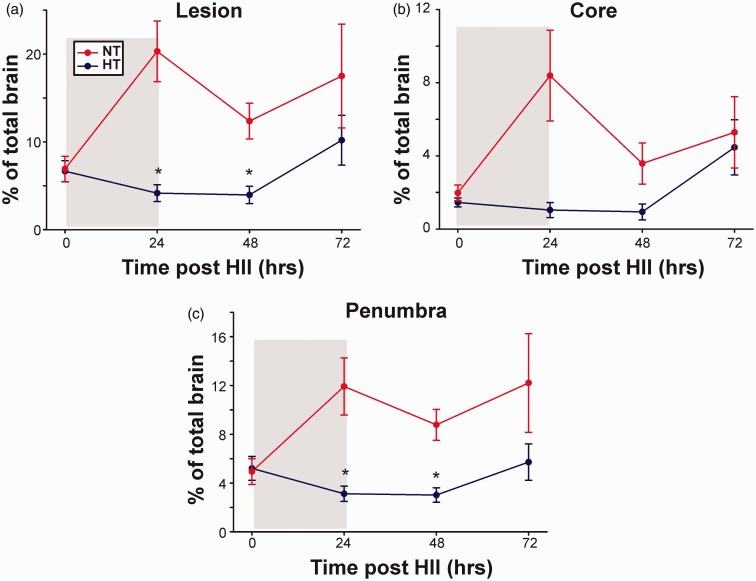
Temporal evolution of HRS-derived HII lesion volumes (total, core, and penumbra). Percent volume of (a) total lesion and individual components of (b) core and (c) penumbra for all HT-/NT-treated pups. HT/NT treatment (24 hr post-HII) is shown in gray. Treatment-based group effects (over 72 hr) were significantly different for total lesion and penumbra (*p* < .05) but not for the core (*p* = .09). HII = hypoxic ischemic injury.

Figure 3(a) to (c) summarizes injury evolution data over time in the NT and HT pups. Lesion volumes were similar immediately after HII (at 0 hr; *p* = .89). Over the entire time period of the study (i.e., 72 hr), HT significantly reduced total lesion and penumbra injury volumes (*p* < .05), but not for the ischemic core (*p* = .09). At 24 hr post-HII (i.e., end of treatment), HT pups had reduced total lesion (80%; *p* < .05) and penumbra (74%; *p* < .05) volumes compared with NT pups, and a tendency toward a reduction in core volume (88%; *p* = .07). This significant HT-mediated decrease in lesion volume (total and penumbra) was maintained up to 48 hr post-HII (*p* < .05) as did the trend toward reduced core volume (*p* = .09). However, between 48 and 72 hr post-HII (i.e., 24–48 hr after discontinuation of HT), we observed increasing trends in total lesion, penumbra, and core volumes in both groups. Although the increase in total lesion volumes was similar in both groups (NT, 12% to 17%; HT, 4% to 10%), we observed that the increase in injury was more prominent in the ischemic core in HT compared with NT pups than in the penumbra. At 72 hr post-HII, total lesion volumes showed a trend toward reduction in HT compared with NT pups (42%; *p* = .37) as did the penumbra (53%; *p* = .25) with little change in the ischemic core (16%; *p* = .77).

**Figure 4.**
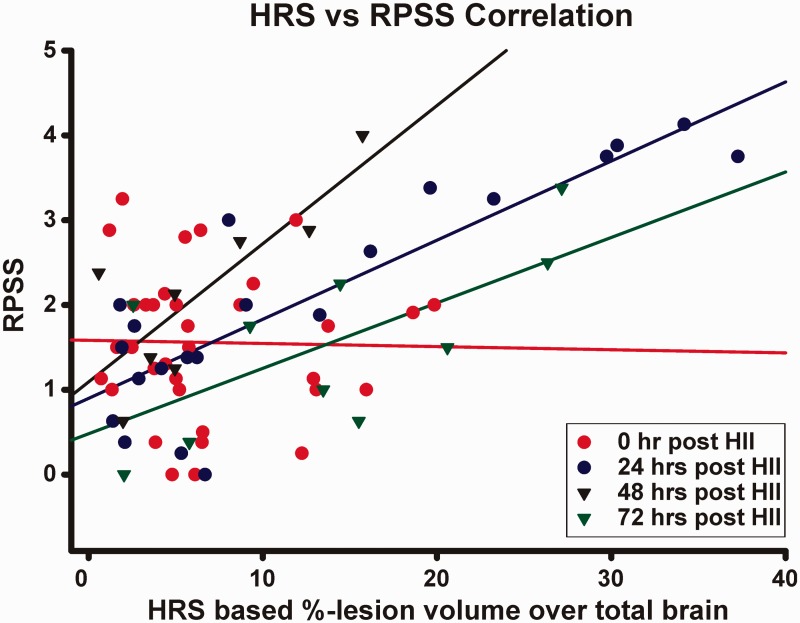
Correlation between RPSS and %-lesion volume: Regional injury-based RPSS data demonstrated good correlation to HRS-derived lesion volumes over all time points (*r*^2 ^= .75), suggesting that both are excellent measures of injury severity. HII = hypoxic ischemic injury; HRS = hierarchical region splitting; RPSS = rat pup severity score.

HRS-derived lesion volumes (% of entire brain volume, BV) and RPSS are different but complimentary measures of injury that were highly correlated (Figure 4) over all time points (*r*^2 ^= .75). The highest correlation was at 24 hr (*r*^2 ^= .86), the time point for which the RPSS method was originally developed; the lowest correlation was at 0 hr (*r*^2 ^= .63), when MRI (ADC) may not accurately reflect injury volume.

Restricted diffusion due to presumed cellular swelling is visible as hypointense ADC within the lesion, and the number of pups exhibiting restricted diffusion at 24 hr post-HII (which is when HT/NT was ended) was the same in both groups (i.e., 100%) as well as at 24 hr post-HT/NT treatment (HT, 80%; NT, 79%). However, at 72 hr post-HII, none of the NT pups exhibited hypointense ADC within the lesion. In contrast, hypointense lesions were still present in 50% of HT pups. Mean ADC values of HII regions (for all data combined) showed no significant differences between the HT/NT groups for hypo- or hyper-intense ADC injury (*p* = .66).

### Cytokine/Chemokine Expression

**Figure 5.**
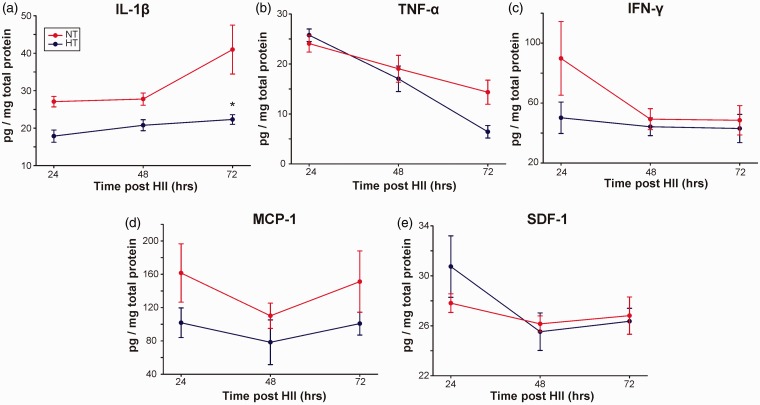
Post-HII expression profiles of brain cytokine/chemokines and signaling molecules. (a) IL-1β was significantly decreased (*p* < .05) in HT compared with NT pups, most prominently at 72 hr post-HII (*: *p* < .05); (b) TNF-α expression level fell significantly faster for HT compared with the NT pups (*p* < .05); (c) IFN-γ had a trend of reduced expression in HT pups compared with NT pups at all time points (*p* = .21), most prominently right after HT treatment; (d) MCP-1 showed a trend toward decreased expression in HT pups at all times points (*p* = .09). (e) SDF-1 was not significantly altered by HT (*p* = .74). HII = hypoxic ischemic injury; HT = hyperthermia; NT = normothermia; IL-1β = interleukin-1β; TNF-α = tumor necrosis factor-α; IFN-γ = interferon-γ; MCP-1 = monocyte chemoattractant protein-1; SDF-1 = stromal cell-derived factor-1.

Expression of the proinflammatory cytokine, IL-1β, was significantly decreased in HT compared with NT pups over the 24 hr to 72 hr post-HII (*p* < .05; Figure 5(a)) but for individual time points, only reached post-hoc significance at 72 hr. TNF-α levels decreased between 24 and 72 hr in both groups, and the rate of decrease was greater in HT pups (*p* < .05). At 72 hr, TNF-α levels in HT pups tended to be lower but this did not reach significance (*p* = .10; Figure 5(b)). There were no significant differences of IFN-γ expression in NT versus HT pups at any time point (*p* = .21; Figure 5(c)). The chemokine, MCP-1, had a trend of reduced expression after HT at all time points (*p* = .09; Figure 5(d)). HT did not alter expression of SDF-1, a signaling molecule involved in stem cell migration (*p* = .74; Figure 5(e)).

**Figure 6.**
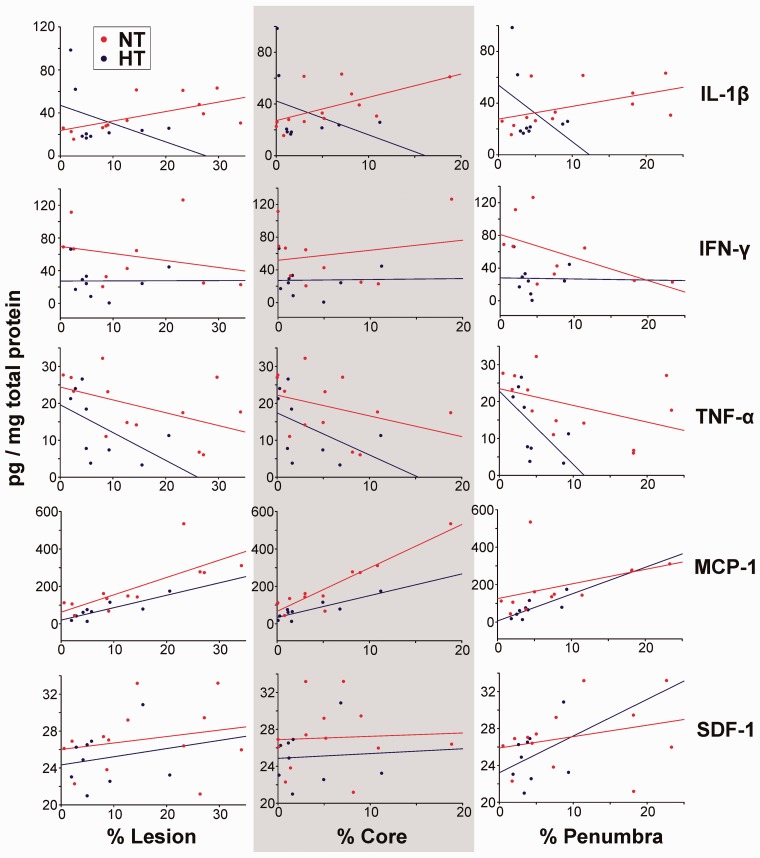
Regression analysis of cytokine/chemokines and signaling molecules relative to lesion volumes. For pups with comparable lesions, core and penumbra sizes (% volumes over the total brain), expression levels of IL-1β, IFN-γ, TNF-α, and MCP-1 were low in HT-treated pups compared with NT pups, while SDF-1 levels were relatively unchanged by HT/NT treatment. TNF-α expression levels over penumbra sizes was more greatly reduced in HT pups (*r*^2 ^= −.34) than in NT pups (*r*^2 ^= −.19). MCP-1 expression levels had contrasting rates of increase between HT versus NT pups and between core and penumbra volumes. SDF-1 expression was comparable in HT (*r*^2 ^= .28) and NT (*r*^2 ^= .19) groups for similar lesion and core volumes, while increasing marginally faster in HT pups (*r*^2 ^= .12) than in NT pups (*r*^2 ^= .08) based on penumbra size. HT = hyperthermia; NT = normothermia; IL-1β = interleukin-1β; TNF-α = tumor necrosis factor-α; IFN-γ = interferon-γ; MCP-1 = monocyte chemoattractant protein-1; SDF-1 = stromal cell-derived factor-1.

Considering only those protein expression data where corresponding MRI data were also available, scatter plots of lesion, core, and penumbra volumes compared with cytokine/chemokine protein levels (merged for all time points) demonstrated that expression levels of IL-1β, IFN-γ, TNF-α, and MCP-1 trended toward reduction in HT pups compared with NT pups, while SDF-1 levels were similar in both groups (Figure 6). IL-1β levels increased with increasing lesion volume in the NT group (*r*^2 ^= .40) but were negatively correlated in the HT group (*r*^2 ^= −.15). IFN-γ did not show any convincing regression trends over injury volumes. TNF-α expression decreased with increase in lesion volume and was more rapid in HT-treated pups (HT, *r*^2 ^= −.28; NT, *r*^2 ^= −.23), and this effect was further accentuated when only penumbra volumes were correlated (HT, *r*^2 ^= −.34; NT, *r*^2 ^= −.19). In contrast, MCP-1 protein levels increased with increasing lesion volume in both HT (*r*^2 ^= .70) and NT (*r*^2 ^= .57) groups. Interestingly, opposite rates of increase were observed in HT and NT pups when MCP-1 was correlated with CP volumes separately. MCP-1 levels in HT pups showed a more rapid increase in the penumbra (*r*^2 ^= .58) compared with the ischemic core (*r*^2 ^= .76). In contrast, MCP-1 levels in NT pups had slower rate of increase in the penumbra (*r*^2 ^= .18) than in the core (*r*^2 ^= .88). For pups with comparable lesions and core or penumbra sizes, HT demonstrated a tendency to lower the expression levels of MCP-1 and IFN-γ compared with NT pups, while SDF-1 levels were comparable between HT/NT. It was noted that, while SDF-1 levels vary similarly (for HT/NT) over the size of the core, in contrast, for increasing penumbra size, SDF-1 expression had a marginally faster increasing trend in HT pups (*r*^2 ^= .12) than in NT pups (*r*^2 ^= .08).

## Discussion

The principal findings of the current study are that HT for 24 hr in a rat pup model of unilateral neonatal HII significantly reduced initial total lesion volume and IL-1β levels compared to NT-treated pups. While MCP-1 had a trend toward reduced levels in HT animals, there were no statistically significant differences at individual time points between HT/NT pups. Importantly, the chemokine signaling molecule SDF-1, which is vital for stem cell migration was not altered by HT. Our data are the first to demonstrate that IL-1β and MCP-1 had tendencies to be reduced after HT (compared to NT) early after neonatal rodent HII and support observations that cytokine activation post-HII may contribute to injury evolution. This supports the generally held concept that HT can inhibit the activity of specific cytokines and may explain its neuroprotective role early after injury (Vexler and Yenari, 2009; McAdams and Juul, 2012). We also observed that HT neuroprotection was mediated primarily by reducing penumbral injury, further supporting the notion that rescuable tissue may be susceptible to inflammatory exacerbation (Figure 3(c)). Combining serial noninvasive imaging with cytokine and chemokine assays during the evolution of injury cascades offers a useful translational model to examine temporal *cause and effect* relationships.

### Hypothermia

Because of its neuroprotective effect, HT is the clinical standard of care for neonatal HII (Shankaran et al., 2012). It also is effective to some degree in adults with global HII who remain comatose following cardiac arrest. A pediatric HT/cardiac arrest clinical trial is near conclusion. In contrast, clinical HT trials in pediatric and adult traumatic brain injury have not shown improvement despite substantial preclinical translational supportive data (Ma et al., 2013). HT is beneficial in adult stroke models with promising but limited evidence of clinical improvement (Zhang et al., 2013). These response differences suggest that HT neuroprotection is related to the underlying injury mechanism(s) as well as the presumed HT reparative processes. Putative mechanisms contributing to HT neuroprotection include (a) suppressed inflammation, (b) reduced excitatory amino acid release, (c) reduced free radical release, (d) decreased cerebral metabolic rate for glucose/oxygen, (e) reduced high-energy phosphate loss, (f) attenuation of secondary energy failure, (g) decreased nitric oxide production, and (h) reduced neuronal apoptosis (Wu and Grotta, 2013).

### HT Effects on MRI-Derived Neonatal HII Lesions

Multiple translational studies (Covey and Oorschot, 2007), some including MRI (Nedelcu et al., 2000; Wagner et al., 2002) or phosphorous/proton spectroscopy (Nedelcu et al., 2000; Taylor et al., 2002; Wagner et al., 2002) have demonstrated that HT can reduce brain injury in rodent neonatal HII models. As is well known, there is substantial HII/HT literature involving newborn lambs, rodents, and piglets. The primary differences in experimental design in rodent models include the (a) duration of hypoxia (15 to 150 min) to mimic mild versus moderate/severe neonatal HII and (b) the depth (30℃–35℃), duration (3–26 hr), or time interval after HII (0–2 hr) when HT was initiated. Three neonatal rodent studies provided MRI data and will be discussed as they relate to the current investigation.

The earliest report using a 7-day-old rat pup model of unilateral HII (90 min hypoxia) followed by 24 hr HT (30℃) starting immediately after HII, described early increases in ADC values as a manifestation of decreased cytotoxic edema (51% of BV), decreased brain energy metabolism (PCr/Pi), as well as a decreased size of the ischemic core at 24 hr (HT: 11% BV; NT: 45% BV) and at 42 days (HT: 12% BV; NT: 35% BV; Nedelcu et al., 2000). Pups with severe initial injury developed large infarctions despite HT and the ischemic core showed no volume decrease whereas some penumbral recovery occurred. In our study, after 48 hr post-HII, core injury started to enlarge as more penumbral tissues became irreversibly affected. Unique to our study was that we used HRS to more accurately detect and quantify injury evolution in the NT and HT groups (Ghosh et al., 2012). In our NT group, within the first 24 hr post-HII, we saw a dramatic increase in core and penumbral volumes (Figure 3), akin to what is considered ‘reperfusion injury’ as is seen in clinical stroke and in rodent models (Leger et al., 2013). In contrast, in our HT pups, the ischemic core did not increase during HT nor for the first 24 hr post-HT but then showed a trend for a rebound effect where it increased dramatically (Figure 3(b)) whereas penumbral volumes remained relatively stable or increased slightly (Figure 3(c)).

A follow-up study by the same investigative group, using similar experimental protocols (105 min hypoxia, 24 hr of HT, 30℃) reported that HT reduced the final injury size at 6 weeks by 23% with greatest recovery in the hippocampus (21%), striatum (13%), and cortex (11%; Wagner et al., 2002). HT also improved behavioral outcomes (Morris Water Maze test, rotarod, circling) with correlations noted between behavioral performance and regional BVs. Similar to their earlier as well as our current study (Figure 3(a)), HT significantly reduced total lesion volume compared with NT pups until 48 hr post-HII at which point we saw acceleration of ischemic injury. Of importance in this study by Wagner et al. (2002) is that they observed that total lesion size at 12 hr and 24 hr increased during HT. These findings contrast to our data which showed that during and for 24 hr after HT, CP volumes did not increase but that at 48 hr, the core and to a lesser extent, the penumbra increased in volume (Figure 3). These findings suggest that the HT protective effect may be time sensitive. Whether this HT core/penumbral tendency toward a ‘rebound effect’ could be ameliorated by increasing the depth/duration of HT or by adding secondary treatments warrants further investigation.

The third report in 7-day-old rat pups (unilateral HII with 150 min hypoxia) used DWI to identify brain lesions prior to study entry and compared NT (36℃/48 hr) versus four HT groups with two different target temperatures (30℃, 33℃) and two durations of HT (24 hr, 48 hr; Lee et al., 2010). HT pups had lower lactate-plus-lipid levels on proton MR spectroscopy at 7 days and greater residual hemispheric volume and improved rotarod and cylinder tests at 5 weeks. Outcome differences between the four HT groups were not found. Our study provides additional data as we acquired early serial imaging data (0, 24, 48, 72 hr) and quantified total lesion volume (instead of residual hemisphere volume) and also used HRS to quantify ischemic CP (Figures 2(a) and 3). Differentiating core from penumbra by using imaging characteristics of tissue densities as a marker of injury, as in our study, may be equally or more sensitive and specific, as well as it is easier to quantify than currently used methods such as diffusion perfusion mismatch (Ghosh et al., 2012). One of our recent publications has demonstrated how methods such as HRS could potentially be used to examine more subtle thresholds of tissue injury than just examining core versus penumbra (Supplementary Figure 3 in Ghosh et al., 2012). This may be helpful in better quantifying more granular levels of tissue injury and is potentially relevant for neonatal HII, which is considered different than adult HII as there is greater evolving apoptotic/mitochondrial injury in newborns. This injury evolution if quantified rapidly with MRI computational methods could offer a more rational approach to therapy augmentation.

#### Complementary Nature of HRS and RPSS

Lesion volumes derived from HRS highly correlated with the RPSS (Figure 4), particularly at 24 hr post-HII, which is the time point for which RPSS was originally developed (Recker et al., 2009). RPSS measures anatomical involvement of injury without accounting for total injury volume and this might explain why we saw a rebound effect at 72 hr post-HII in HRS-determined lesions (Figure 3(a)), while we did not see the same using the RPSS (Figure 2(d)). HRS quantifies lesion size without designating which brain regions are affected, potentially not accounting for differences in regional severity. For example, if hippocampal injury volume resolves while cortical injury increases, RPSS will decrease while HRS might increase. On the other hand, while HRS can estimate ischemic CP regions, RPSS cannot. We believe that, in the future, complementary data from HRS and RPSS could be utilized in an information fusion model.

Reflecting the findings in rat pup studies, several recent clinical studies have demonstrated MRI evidence of reduced brain injury in HII neonates treated with HT compared with untreated HII neonates. Reduced injury (in HII neonates after HT), in cortical gray and white matter, basal ganglia, thalamus, and the posterior limb of the internal capsule has been reported (Rutherford et al., 2010), as well as a higher percentage of neonates with a normal MRI after HT (52%) compared with NT (35%) and a lower incidence of infarction (HT, 12%; NT, 22%; Shankaran et al., 2012). Additional studies reported serial imaging solely in HT-HII neonates (i.e., no NT controls) with two important observations. First, it was found that HT could be safely and reproducibly maintained during MRI acquisition, suggesting that obtaining scans during treatment could better estimate the severity of injury allowing clinicians the opportunity to consider additional treatments. In addition, when MRI was acquired 2 to 3 days post-HII, it was noted that lesions present at this early time point likely were irreversible (i.e., ischemic core; Wintermark et al., 2011). Such findings highlight the need for using more clinically relevant animal models to explore the imaging and biochemical temporal evolution of injury as in the current study. As the ability to serially image seriously ill newborns becomes more readily and safely available, similar methods would be more translatable to future clinical research, for the evaluation of candidate selection for treatment and for early outcome assessment of specific combinatorial therapies.

### HII, HT, and Cytokine/Chemokine Interactions

#### Effects of HII on cytokines/chemokines in adult and neonatal models

##### IL-1

IL-1β mRNA elevations have been documented within 15 to 30 min after ischemia in adult HII models with increased protein within several hours (Davies et al., 1999). Following 20 min of rodent transient global cerebral ischemia, IL-1β mRNA and protein expression is increased early during reperfusion (1 hr), but also at later times (6–24 hr), indicating biphasic expression (Alonso-Alconada et al., 2012). Consistent with the injurious role of IL-1, administration of IL-1β in rats results in increased brain damage (Yamasaki et al., 1995). Also, wild-type mice have larger infarcts compared with those deficient in IL-1. Our data (Figure 5(a)) support the potentially injurious role of IL-1β.

Overexpression or treatment with IL-1 receptor antagonist (IL-1ra) protein can reduce infarct size (Yang et al., 1997), while IL-1ra deficient mice exhibit a dramatic increase in ischemic damage (Pinteaux et al., 2006). In addition to numerous studies that have shown increased IL-1β after HII, inhibition of IL-1β activity (with IL-1 receptor blocking antibodies or by activation of IL-1-converting enzyme) has resulted in decreased brain injury in neonatal HII models (Clarkson et al., 2005; Girard et al., 2012). Thus, IL-1 appears to exacerbate cerebral injury but this may depend on the subtype.

##### TNF-α

In our study, we found that TNF-α levels were elevated and then slowly and similarly decreased in NT and HT pups over 72 hr, although significantly (*p* < .05) greater in HT-treated pups (Figure 5(b)). TNF-α also has been shown to be upregulated after cerebral ischemia with similar expression patterns as IL-1β. Initial increases are seen 1 to 3 hr after ischemia (Liu et al., 1994), and similar to IL-1β, there is biphasic expression with a second peak at 24 to 36 hr (Offner et al., 2006). TNF-α expression was initially observed in neurons (Liu et al., 1994), then later in microglia and in some astrocytes as well as in the peripheral immune system (Offner et al., 2006). TNF-α appears to have pleiotropic functions in the ischemic brain (Hallenbeck, 2002). Inhibition of TNF-α reduces ischemic brain injury (Yang et al., 1997), while administration of recombinant TNF-α protein after stroke onset worsens ischemic brain injury, although under certain circumstances TNF-α may be neuroprotective (Barone et al., 1997).

##### MCP-1 and SDF-1

Multiple chemokines such as MCP-1 can be induced in animal models of focal cerebral ischemia (Chen et al., 2003). Consistent with a deleterious role, overexpression of MCP-1 in the brain exacerbates ischemic injury and correlates with inflammatory cell recruitment. The importance of MCP-1 in neonatal HII, also has been demonstrated as IL-1 converting enzyme in knockout mice (which have previously been shown to be less susceptible to HII), attenuated the MCP-1 increase which suggested that MCP-1 induction is essential for HII neuronal injury (Xu et al., 2001). In addition to its chemotactic properties, MCP-1 directly affects blood–brain barrier (BBB) permeability. Addition of MCP-1 resulted in a 17-fold increase in the permeability of an *in vitro* BBB model (cocultures of endothelial cells and astrocytes) and caused alterations in tight junction proteins, suggesting that MCP-1 may play a role in “opening” the BBB (Stamatovic et al., 2005). With the interest in stem cell-based therapy for ischemic brain injury, chemokines appear to function as stem cell signaling molecules and play an important role in attracting stem cells to ischemic regions (Newman et al., 2005). MCP-1 and SDF-1 and their receptors have been observed at the interface of ischemic tissue and cell transplants (Kelly et al., 2004). MCP-1 also seems to be involved in marrow-derived stromal cell migration into ischemic brain (Wang et al., 2002).

#### Effects of HT on cytokine/chemokine activity in models of neonatal HII:

##### Cytokines

Data from other investigators as well as our current study (Figure 5) support a role for HT inhibiting cytokine-mediated postneonatal HII toxicity (Fukui et al., 2006; Xiong et al., 2009). HT has variable effects on postinjury cytokine release and activity depending on the model used, site, timing of measurement, and the presence of infection. As in our current study (Figures 3(a) and 5(a) to (d)), the majority of neonatal unilateral HII studies found that HT decreased infarct volume as well as cytokine expression (IL-18, IL-6, and TNF-α), decreased microglial activation, and improved behavioral outcome (Figure 2(c); Fukui et al., 2006; Xiong et al., 2009).

Our study is the first to show that HT reduced IL-1β (*p* < .05) and had a trend to reduce MCP-1 (*p* = .09) after neonatal HII (Figure 5). As noted earlier, there is a biphasic (early, 1–3 hr; late, 24–36 hr) increase in cytokine expression although in our NT pups the late increasing trends were observed at 72 hr post-HII for both IL-1β and MCP-1 (Figures 5(a) and (d)). Of interest is that after HT, IL-1β expression was similar across all time points, irrespective of the degree of corresponding NT levels (*p* < .05).

We also observed dissociation between the late increase in HII volumes at 72 hr (Figure 3) when there were trends of considerable inhibition of IL-1β and MCP-1 (Figures 5 and 6), raising the question as to the nature of the underlying mechanisms responsible for this paradoxical effect. As the increase in lesion volume was mainly due to core expansion (Figure 3), it is possible that there were differences in core versus penumbral IL-1β and MCP-1 expression in which core levels remained high whereas penumbral levels continued to decrease. Because we used the entire HII hemisphere tissue, we were unable to address this possibility. It also is possible that there are other injury cascade pathways which are activated at later coinciding time points to accelerate injury despite the lower cytokine levels. Interestingly, delayed diffusion reversal was observed in the HT-treated pups where ADC hypo-intensity was present even at 72 hr post-HII, while such hypo-intensity was not noted in the NT pups. This result and similar recent reports on the effect of HT in term neonates with hypoxic-ischemic encephalopathy (Bednarek et al., 2012) also might suggest another possibility, that HT neuroprotection probably delayed the post-HT increase in IL-1β and caused a tendency of blunting of the late increase in MCP-1 (Figure 5). This possibility in HT-treated pups is further supported by the tendencies of a late increase in CP volumes (Figure 3) and a late decrease in weight gain and righting reflexes (Figure 2(b) and (c)) at 72 hr post-HII. Finally, the rewarming phase after HT is gaining increased attention not only as a possible contributor to injury but also as a neuroreparative mechanism in which anti- (instead of pro-) inflammatory cytokines are released (Diestel et al., 2010; McAdams and Juul, 2012) and warrants further study.

##### Chemokines

MCP-1 functions as both a cytokine and chemokine, whereas SDF-1 is primarily a chemokine involved in chemoattractant functions associated with stem cell migration (Imitola et al., 2004). Injury progression in NT pups is rapid perhaps due to evolving vasogenic edema that is not only reduced in HT pups (Figure 3) but its appearance was delayed too, as reflected in delayed diffusion reversal in ADC data. Close review of MCP-1 correlations with injury volumes (Figure 6) shows trends that MCP-1 is reduced in HT but increases more dramatically in animals with larger penumbra volumes. All results and trends from this study intuitively suggest that HT is able to reduce but not eliminate anti-inflammatory cytokine toxicity and support the idea that HT is neuroprotective, potentially by extending the treatment window after HII (Bednarek et al., 2012). Thus, an additional intervention like stem-cell therapy could extend and sustain the effects of neuroprotection. We were interested in determining whether HT conferred a differential effect on chemokines because of the concern that HT might, in a time-dependent manner, blunt SDF-1 (or other signaling molecules) that could affect the optimal activities of stem cells after neonatal HII. Our data demonstrated trends that HT downregulated MCP-1 expression (although it failed to reach statistically significance; *p* = .09) but not SDF-1 (Figures 5(d) and (e)). Moreover, HT demonstrated a weak trend (*r*^2 ^= .12) of increasing SDF-1 expression with an increase in penumbra size in HT pups (Figure 6). As HT is standard of care, this is an important consideration as use of stem cell transplantation would only be done as a combinatorial therapy with HT. As with many other neuroprotectants, careful examination of the timing after HT will be necessary to maximize benefit.

## Conclusions

HT (32℃; 0–24 hr post-HII) reduced neonatal HII lesion volume (*p* < .05) in the acute stage (0–48 hr post-HII), primarily by reducing penumbral injury (*p* < .05) and the proinflammatory cytokines IL-1β (*p* < .05) and by trends of reducing MCP-1 (*p* = .09) without affecting the stem cell migrational signaling molecule SDF-1. Combining serial imaging with assays of biologically activated molecules could improve methods to selectively develop new therapies. Although extensive validation is needed, our data suggest that HT, if used in combination with stem cell implantation, would not adversely affect signaling pathways that serve as attractants that foster migration toward neonatal HII lesions. Hopefully, this could provide additive neuroprotection, contribute to tissue repair, and improve functional outcome.

## Summary

Combining serial neuroimaging with assays of activated cytokines and chemokines in a neonatal hypoxic ischemic rat model, we found that hypothermia reduced early lesion volume primarily by reducing penumbral injury and the proinflammatory cytokine IL-1β, without affecting stem cell migrational signaling molecules.
